# Assessment of personality disorders in adolescents – a clinical validity and utility study of the structured interview of personality organization (STIPO)

**DOI:** 10.1186/s13034-025-00901-9

**Published:** 2025-05-02

**Authors:** C. Laczkovics, K. Czernin, A. Bründlmayer, M. Zeiler, W. Bangerl, C. Prause, P. L. Plener, S. Doering, V. Blüml, J. Carlitscheck, S. Bender, M. Krischer

**Affiliations:** 1 Department of Child and Adolescent Psychiatry, Medical University of Vienna, Währinger Gürtel 18-20, 1090 Vienna, Austria; 2 Department of Child and Adolescent Psychiatry and Psychotherapy, University of Ulm, Ulm, Germany; 3 Medical Faculty, Department of Child and Adolescent Psychiatry, University of Cologne, Cologne, Germany; 4 Department of Child and Adolescent Psychiatry, University Clinic Cologne, Cologne, Germany; 5 Department of Psychoanalysis and Psychotherapy, Medical University of Vienna, Vienna, Austria

## Abstract

**Background:**

The diagnosis of personality disorders (PD) in adolescence still poses a challenge. Early diagnosis and targeted intervention are called for, since patients with PD present with severe consequences in terms of psychosocial functioning and personal suffering including higher suicide risk. New guidelines advise semi-structured interviews for the dimensional assessment of personality functioning.

**Methods:**

We included 136 patients aged 13 to 17.9 years with a categorical PD diagnosis and 35 healthy control (HC) adolescents to assess the applicability of the Structured Interview for Personality Organization (STIPO) for adolescents and evaluate its validity by correlating the six outcome domains (identity, object relations, defenses, aggression, moral values, reality testing) and the overall severity level to several validated instruments. Furthermore, we assessed personality traits, internalizing and externalizing behavior and depressive symptoms.

**Results:**

All STIPO domains differed significantly between patients and HC (*p* <  0.001). Outcome measures correlated significantly with validated self-rating questionnaires. STIPO severity levels correlated significantly with psychopathology. Personality traits “dissocial behavior” and “emotional dysregulation” correlated positively with all STIPO domains and the overall level of personality organization (PO).

**Conclusions:**

Results indicate that the STIPO is a reliable and valid instrument for the assessment of PD in adolescents. It comprises the core elements of personality functioning, as requested in Criterion A in the AMPD of the DSM-5 and ICD-11 and could be useful for treatment planning, evaluation of the course of treatment as well as for prognostic considerations.

**Supplementary Information:**

The online version contains supplementary material available at 10.1186/s13034-025-00901-9.

## Background

After decades of negligence to diagnose personality disorders (PD) in adolescence, because of its stigmatizing nature and the notion of incurability [1], it has nowadays been increasingly acknowledged that PDs do occur in children and adolescents [2–4]. Early diagnosis is called for, since patients with PD present with severe consequences in terms of psychosocial functioning and personal suffering [5] including higher suicide risk [6]. Targeted early intervention might lead to better outcome [7]. Nevertheless, the diagnosis and differential diagnosis of PD in adolescents represents a challenge [8]. Adolescence is a period of rapid change and a crucial phase for the development and consolidation of identity [9–11]. Changes in brain architecture lead to risk-taking/novelty-seeking behavior, as do the hormonal changes of puberty [12]. Furthermore, the developmental task of separation from the primary caregivers and orientation towards the peer group can further increase risky behavior as well as emotional instability. Low self- directedness and instability of one´s self-esteem are common.

The traditional diagnostics of personality disorders in DSM-5 [13] and ICD-10 [14] utilizing a categorical approach based on symptomatic manifestations are therefore not the via regia to diagnosis [15]. In contrast, the new alternative model for PD (AMPD) in the DSM-5 [13] as well as the ICD-11 section on PD [16] advocate a dimensional approach and focus on personality functioning [17, 18]. The German national guideline advises to diagnose PD in adolescents of 12 years and older using interviews for dimensional assessment rather than using a categorical approach or self-report instruments [19]. For the diagnosis of PD an impaired functioning of aspects of the self (e.g., identity, self-worth, accuracy of self-view, self-direction), and/or of interpersonal relations (e.g. ability to develop and maintain close and mutually satisfying relationships, ability to understand others’ perspectives and to manage conflicts in relationships) is required. This dimensional approach that focuses on underlying personality functioning rather than the occurrence of a certain symptomatology might be the most appropriate for diagnosing PD in adolescence [20, 21].

Semi-structured interviews for the new dimensional approach in adolescents are the focus of recent research [22], but literature on assessment tools is still scarce [23–25]. To our knowledge, only the study by Thompson et al. studied adolescents with severe impairment in psychosocial functioning and a categorical PD diagnosis [25].

These considerations converge with long standing psychoanalytic conceptualizations of personality structure or organization (PO), respectively [18]. One influential contemporary model to conceptualize PO was developed by Otto Kernberg who proposed three basic levels of PO: neurotic, borderline, and psychotic PO [26–29]. These levels of PO are distinguished by differences in the areas of identity integration, maturity of defense mechanisms, and the capacity for reality testing. Neurotic PO is defined by an integrated view of the self, with distinct boundaries to others, the capacity for close relationships and intact reality testing. The most severe impairment is found in psychotic PO that is defined by impaired reality testing. Borderline PO (BPO) is specified by intact reality testing but impairment of the view of self and others. For the reliable diagnosis of a specific level of PO Kernberg and colleagues developed the “Structured Interview for Personality Organization” (STIPO; [26, 30, 31] that assesses various dimensions of personality functioning.

The STIPO is a validated instrument for the assessment in adults, and we hypothesize that it is a probate assessment interview for determining personality functioning in adolescents as well. The STIPO enables for the dimensional assessment of the overall level of PO on six specified levels (ranging from normal to severely impaired PO). It also provides a detailed evaluation of the level of identity integration comprising the assessment of the sense of self and the sense of others, as well as interpersonal relating. Therefore, the information obtained by the STIPO directly concerns the core elements of personality functioning identified by the AMPD of the DSM-5 and ICD-11 [18, 29]. With this study we furthermore introduce the revised and shortened version of the STIPO, the STIPO-R [32] that we adapted for adolescent language, the STIPO-R-A. We performed a clinical utility and validity study by correlating the outcome domains to several validated instruments for adolescents as well as psychopathology measures.

After assessing personality functioning, Criterion B of the AMPD gives the option to specify pathological personality traits. Five domains are listed that are aligned with the Five Factor Model of personality and assessment inventories have been developed [33]. In our study, we used the “Dimensional assessment of personality pathology-basic questionnaire” (DAPP-BQ [34]) that is validated in adolescents [35] and was reported to have a high overlap with the Five Factor Questionnaires established for the ICD-11 [36]. We hypothesized that pathological personality traits correlate with PO. As the constructs are different, we further hypothesized that the correlation would be lower than the correlation of instruments regarding personality structure and functioning as the constructs are different [37].

## Methods

### Sample

We included 136 patients aged 13 to 17.9 years with a probable or definite PD diagnosis according to the International Personality Disorder Examination (IPDE) [38, 39]. Depending on the specific PD, at least 3–4 criteria were met. This decision was made acknowledging that researchers in adolescent psychiatry have suggested that the worst solution was to consider probable PD as no PD [1]. They confirmed that sub-threshold cutoff points show a better fit for adolescents. Furthermore in treatment studies, adolescent researchers use cutoff points of 3 as well, considering the clinical necessity to treat these adolescents with specialized protocols for PD and underscoring the clinical value of including probable PDs to a clinical sample of PD [2]. Moreover, we intended to include patients with a wide range of level of personality functioning to address our research question on dimensional assessment of adolescent PD. Further inclusion criteria were sufficient knowledge of the German language, no intellectual disability and absence of psychosis and acute suicidality. After informed consent had been obtained from patients and their legal guardians, patients underwent the interview process and filled out self-rating questionnaires. In study center 1 [University Hospital of X, *blinded for review*], 69 patients were screened for eligibility. Twelve patients had to be excluded (two, due to missing values and ten because they did not meet criteria for PD in the IPDE), leaving 57 in the study. In study center 2 [Day Clinic of a University Hospital of Y, *blinded for review*], 79 patients, that had a probable or definite PD diagnosis in the IPDE, were screened for eligibility and were included. No patient had to be excluded. Thus, the total sample consisted of 136 patients.

We recruited age and sex matched healthy control persons (HC) from local schools, attending grades nine and ten, in study center 1. HC could take part in the study if they were not in psychiatric treatment at the time, had sufficient knowledge of the German language and average intellectual abilities. After informed consent was obtained from the participant and their legal guardian, they underwent the interview process and filled out self-rating questionnaires. 35 HC volunteered and were included in the analysis after exclusion of one person due to missing values.

The study was approved by the local ethics committee of study center 1 (EK Nr.:1174/2021) and study center 2 (EK Nr.:21-1129_1, DRKS 0010557).

### Measures

The following standardized interviews and questionnaires were used.

#### Structured interview of personality organization, original version (STIPO) and revised version adapted to the adolescent language (STIPO-R-A)

The STIPO [30, 32] is the structured version of the Structural Interview [27, 42].

The original interview consists of 100 items, for the revised version redundant questions were removed as well as the domain “reality testing”. For the use in adolescence, we adapted the questions to adolescent language and kept the domain “reality testing” due to its clinical importance in adolescent psychopathology.

The newly revised version, adapted to adolescent language (STIPO-R-A) consists of 63 items that are addressed by one or more specific questions. Forty-eight patients were rated with the original version of the STIPO, 88 patients and all controls were rated with the STIPO-R-A. The single-item rating is performed by the interviewer on a three-point scale with operationalized descriptions for each level: 0 = pathology absent; the trait being queried is not present at all, or if slightly present has no impact on respondent’s functioning. 1 = the trait being queried is present and reflective of some pathology, sub-threshold; minor impairment. 2 = the trait being assessed is clearly present, and reflects significant to severe pathology; significant to severe impairment.

The STIPO assesses six domains and sub-domains, see Table 1.

**Table 1:** Domains and subdomains of the STIPO-R-A

Domain (number of questions)	Subdomain	Example questions
Identity (15)	Capacity to invest in work/studies and recreation Sense of self Sense of others	“How capable are you at school?” “Tell me about yourself, what are you like as a person? Let’s say that you wanted me to get to know you as quickly as possible, in just a few minutes– how would you describe yourself to me so that I get a lively and well-rounded picture of the kind of person you are?“
Object relations (14)	Interpersonal relations Intimate relationships and sexuality Internal working model of relationships	“Are your closest friends people with whom you can share the more intimate details of your life, your successes and joys, as well as your disappointments, difficulties and fears?“ “Are there significant things that those close to you do not know about you, or that they would be quite surprised to learn about you?“
Defenses (13)	Lower-level, primitive defenses Higher-level defenses, coping	“Do you tend to look up to people, to put them on a pedestal?” “Do you tend to see yourself or others, or situations, in black and white or in all-or-nothing terms?” “When plans that you are counting on fall through, or when you spend a great deal of time and effort planning for a situation only to find that the plans fall through, how do you typically respond?“
Aggression (9)	Self-directed aggression Other-directed aggression	“Do you hurt yourself when you are in trouble?” “Do you lose your temper with others?”
Moral values (6)	Experience of guilt Moral and immoral behavior	“Are you confused about what to do when it comes to things like lying, stealing, or cheating if you think you can get away with it?“ “Have you ever done anything that is illegal?”
Reality testing (6)		“Are there times when you have a sense you are not your usual self, or that you are so disconnected from your usual experience that you feel estranged from yourself and the surrounding world?”

The STIPO-R-A also includes an overall rating of narcissism, consisting of different items out of the assessed dimensions which also indicate the presence of narcissistic features.

Each domain and subdomain are rated on a five-point scale with “1” representing the absence of pathology and “5” indicating most severe impairment. Operationalized guidelines exist for each rating. Finally, an overall rating is generated from the ratings of the dimensions. Six different levels of PO (i.e. personality functioning) are provided for the overall rating: [1] normal [2], neurotic 1 [3], neurotic 2 [4], borderline 1 [5], borderline 2, and [6] borderline 3.

Satisfactory reliability and validity of the English as well as the German version of the STIPO have been demonstrated for adults [31, 43]. In the German version, Interclass Correlation Coefficients (ICC) were 0.89− 1.0, and Cronbach´s α were between 0.93 for identity, 0.69 for reality testing and 0.97 for the level of PO.

STIPO-interrater reliability between the raters was assessed after an intensive STIPO training under the supervision of a senior STIPO-scholar and was based on six videotaped training cases which were not part of this study. ICC were calculated in SPSS with a two way mixed model with single measure and absolute agreement with mostly good to excellent results [44]. Results for all STIPO domains across raters from both study centers (12 raters): Identity: 0.83, Object relations: 0.85, Primitive defenses: 0.69, Coping: 0.67, Aggression: 0.85, Moral values: 0.53, Reality testing: 0.84, level of PO: 0.85.

#### International personality disorder examination (IPDE) [38, 39]

The IPDE is a structured interview for the diagnosis of PDs according to DSM-5 and ICD-10. In the primary section, the history of life and disorder is assessed in a free manner. This is followed by the structured interview part, and starts with more superficially available topics regarding work and school and proceeds to more difficult areas like sexuality and delinquent behavior. The IPDE used in this study consists of 99 semi-structured questions assessing the 11 PDs defined by the DSM-4. There are three possible outcomes for PD according to the IPDE: positive, probable or negative. Full-syndrome BPD and sub-threshold BPD show difficulties on the same spectrum, therefore we included definite as well as probable PD diagnosis to the clinical sample in line with prior studies [40, 41, 45] and in consideration of adolescent age. In a worldwide study, the inter-rater reliability showed excellent agreement among the examiners, also test-retest reliability was high [39].

#### Inventory of personality organization-adolescents (IPO-91-A) [46–49]

This self-rating instrument consisting of 91 items is based on the theoretical structural model by Kernberg and investigates the severity of the domains “identity diffusion”, “aggression”, “reality testing”, “primitive defenses”, “moral values” and “instability of goals”. Each item has to be rated on a five-point scale ranging from “Never true” to “Always true”. The internal consistency was α = 0,90.

#### Operationalised psychodynamic diagnosis in children and adolescents - structure questionnaire (OPD-KJ2-SF) [50]

This self-report instrument measures the four dimensions of personality structure: “Self-direction”, “identity”, “interpersonality” and “attachment” in adolescents between 12 and 18 years. It contains 81 items on a 5-point Likert scale ranging from 0 (not true) to 4 (very true). Psychometric properties were good, with Cronbach´s α ranging from 0.87 to 0.98. This questionnaire was completed by participants from study center 1 only.

#### Levels of personality functioning questionnaire, LoPF-Q 12–18 [51]

The LoPF-Q 12–18 is a self-report questionnaire for adolescents between 12 and 18 years to assess the dimensions of personality functioning according to Criterion A in the AMPD of the DSM-5: “Identity”, “self-direction”, “empathy”, and “intimacy”. It enables a dimensional differentiation between healthy and impaired personality functioning which is assumed to be associated with high risk for a current PD. It contains 97 items with a 5-step answering format (0 = no to 4 = yes). The four resulting primary scales are each composed of two subscales. In addition, a total score is calculated to quantify a general severity level of functional impairment. The questionnaire showed good scale reliabilities and good construct validity. Again, data from this measure are available from participants recruited from study center 1 only.

#### The dimensional assessment of personality pathology—basic questionnaire DAPP-BQ [34, 35, 52]

The DAPP-BQ is a 290-item self-report measure with five response categories for each item. The DAPP measures 4 personality traits, “emotional dysregulation”, “dissocial behavior”, “inhibitedness” and “compulsivity” that comprise 18 factors.

Internal consistency ranges from 0.80 to 0.94 and test-retest reliability over a three-week period ranges from 0.81 to 0.93 [53]. The German version of the DAPP-BQ was developed using a forward-backward translation method which has been tested for its factorial validity in clinical and nonclinical samples [53, 54].

#### Beck’s depression inventory-II [55]

The BDI-II is a self-rating questionnaire which assesses the severity of depression. It is validated for adolescents from age thirteen to nineteen and for adults. The BDI-II includes twenty-one items. Every item consists of 4 statements which stand for a different severity of depression. After completing the BDI-II it is possible to build a global score. A BDI-score of 14 or higher is considered as clinically relevant. Cronbach´s α is between 0.90 and 0.93 [55].

#### Youth self-report [56]

The YSR is a self-rating questionnaire for 11- to 18-year-olds to assess psychiatric problem areas. It consists of 99 items with three respond categories. It measures the extent of internalizing and externalizing problems. The scale for internalizing problems consists of three syndrome scales: social withdrawal, somatization and anxiety/depression. The scale for externalizing problems consists of two syndrome scales: delinquent behavior and aggressive behavior. Additionally, a total score was calculated summing up item ratings from the internalizing and externalizing scales as well as additional subscales (social problems, thought problems, attention problems). For the German version, internal consistency (> 0.90), test-retest reliability (0.86–0.90) and factorial validity was found to be satisfying to good [57]. The raw scores were transferred to T-Scores according to the available German norm sample. For the YSR overarching scales, a T-Score ≥ 64 and for the syndrome scales a T-score ≥ 71 are considered clinically relevant.

### Statistical analysis

The statistical analyses were performed using IBM SPSS Statistics 29.0 and JASP 18.0 software. First, we descriptively compared sociodemographic and clinical characteristics of the patients vs. HC sample and evaluated group differences using t-tests (for continuous variables) and Chi²-tests, respectively Fisher’s exact tests (for categorical variables). We further calculated the prevalence of PD diagnoses according to the IPDE as well as the distribution of levels of PO according to the STIPO for the patient and HC samples. Inter-correlations between the STIPO domains were evaluated using Pearson correlation coefficients. To test the hypothesis that average STIPO ratings are higher in individuals with a definite or probable PD according to the IPDE than in individuals with no PD, t-tests for independent samples were used and Cohen’s d effect sizes were calculated. As differences were calculated for a total of ten STIPO domains, a difference was deemed statistically significant if the p-value was < 0.005 (Bonferroni-corrected alpha level applied).

Moreover, we conducted hierarchical linear regression models to evaluate how much variance in the general psychopathology (measures by the YSR total T-score) can be explained by the categorical personality assessment using the IPDE vs. the dimensional assessment using the STIPO. In the first regression model, we included a set of IPDE outcome variables as predictors in the first step and evaluated whether additionally including STIPO outcome variables in the second step significantly increased the explained variance in general psychopathology (ΔR²). In the second model, we included STIPO outcome variables as predictors in the first step and analyzed whether additionally including IPDE outcome variable significantly improved the model fit.

In order to evaluate the convergent validity of the STIPO domains with other measures of personality and general psychopathology, we calculated Pearson correlation coefficients between STIPO domains and other questionnaire scores. To account for multiple testing, we used a significance level of 0.001 for these correlation analyses. We predefined the following specific hypotheses according to other instruments that have been validated for the use in adolescents [49–51, 53]:

1. We hypothesized that STIPO scores were positively correlated with the IPO-A [49] that is based on the same theoretical concept, Kernberg´s object relations theory.
2. We hypothesized that STIPO scores were positively correlated with the OPD KJ SF [50]. The structure domain of the OPD that is assessed with this questionnaire is based on a common theoretical foundation insofar as it is also a psychoanalytically grounded instrument.
3. We hypothesized that STIPO scores were positively associated with the LoPF-Q [51], that measures personality functioning according to ICD-11 and the AMPD of the DSM-5.
4. We hypothesized that STIPO scores were correlated with pathologic personality traits measured by the DAPP-BQ [53].
5. We hypothesized that STIPO scores were correlated with psychopathologically relevant symptoms measured with the BDI and the YSR.

## Results

### Sample characteristics

A total of 136 patients with a mean age of 15.8 (SD 1.17) years and 35 HC with a mean age of 15.8 (SD 1.11) years were included in the study. Demographics and psychopathology, determined by the YSR and the BDI, are given in Table 2. The patient and HC samples were comparable in age and sex distribution; however, as expected, the patients showed significantly higher scores of different self-rated symptom scales compared to HC.

**Table 2:** Sample characteristics

	Patients (*N* = 136)	Controls (*N* = 35)	Group difference
	Mean (SD)	Mean (SD)	Test statistic, *p*-value
Age	15.83 (1.17)	15.80 (1.11)	*t*(169) = 0.141, *p* = 0.888
BDI total score	34.60 (11.79)	12.88 (9.45)	*t*(159) = 9.796, *p* = < 0.001
YSR total T-score	75.85 (5.48)	69.75 (7.09)	*t*(161) = 5.947, *p* = < 0.001
YSR internalizing T-score	75.26 (6.49)	65.52 (9.07)	*t*(160) = 7.052, *p* = < 0.001
YSR externalizing T-score	60.95 (9.73)	55.71 (7.85)	*t*(161) = 2.904, *p* = 0.002

	n (%)	n (%)	
Sex			
Females	124 (91.2%)	32 (91.4%)	Fisher’s exact test: *p* = 1.000
Males	12 (8.8%)	3 (8.6%)	
Clinically relevant depression^a^	124 (96.9%)	16 (48.5%)	Fisher’s exact test: *p* < 0.001
Clinically relevant psychopathology^b^			
YSR total	126 (97.7%)	28 (82.4%)	Fisher’s exact test: *p* = 0.003
YSR internalizing	119 (92.2%)	17 (51.5%)	χ²(1) = 32.659, *p* < 0.001
YSR externalizing	48 (37.2%)	5 (14.7%)	χ²(1) = 6.210, *p* = 0.013
YSR withdrawn	65 (50.4%)	4 (11.8%)	χ²(1) = 16.443, *p* < 0.001
YSR somatic complaints	51 (39.5%)	7 (20.6%)	χ²(1) = 4.214, *p* = 0.040
YSR anxious/depressed	99 (76.7%)	9 (26.5%)	χ²(1) = 30.419, *p* < 0.001
YSR social problems	27 (20.9%)	0 (0.0%)	χ²(1) = 8.529, *p* = 0.003
YSR thought problems	41 (32.0%)	5 (14.7%)	χ²(1) = 3.966, *p* = 0.046
YSR attention problems	64 (49.6%)	1 (2.9%)	χ²(1) = 24.446, *p* < 0.001
YSR dissocial behavior	24 (18.6%)	1 (2.9%)	χ²(1) = 5.084, *p* = 0.024
YSR aggressive behavior	9 (7.0%)	1 (2.9%)	Fisher’s exact test: *p* = 0.689

^a^BDI-score ≥ 14; *n* = 8 missings in the patient sample, *n* = 2 missings in the control group

^b^T-score ≥ 64 for YSR broadband scale; T-score ≥ 71 for YSR syndrome scales; *n* = 7 missings in the patient sample, *n* = 1 missing in the control group

The group differences of the categorical PD diagnoses with the IPDE are provided in Table 3. Table 3 also presents the PO derived from the dimensional assessment with the STIPO. While all patients had at least one PD diagnosis in the IPDE (as this was defined in the inclusion criteria), also five HC subjects received one or more PD diagnoses. With regard to the STIPO level of PO, the great majority of the patient sample were on levels ‘borderline 1’ and ‘borderline 2’, while in the HC sample, about half of the adolescents were on normal level and the majority of the remaining individuals were on levels ‘neurotic 1’ and ‘neurotic 2’.

**Table 3:** Categorical and dimensional PD diagnosis

	Patients (*N* = 136)	Controls (*N* = 35)
	Mean (SD)	Mean (SD)
IPDE diagnosis (probably and definite)^c^		
Paranoid	33 (16.9%)	1 (2.9%)
Schizoid	6 (4.4%)	0 (0.0%)
Schizotypal	5 (3.7%)	0 (0.0%)
Antisocial	13 (9.6%)	1 (2.9%)
Borderline	102 (75.0%)	1 (2.9%)
Histronic	11 (8.1%)	2 (5.7%)
Narcissistic	10 (7.4%)	2 (5.7%)
Avoidant	90 (66.2%)	2 (5.7%)
Dependent	28 (20.6%)	1 (2.9%)
Obsessive-compulsive	18 (13.2%)	2 (5.7%)
STIPO level of personality organization		
Normal	0 (0.0%)	16 (45.7%)
Neurotic 1	0 (0.0%)	7 (20.0%)
Neurotic 2	2 (1.5%)	10 (28.6%)
Borderline 1	61 (44.9%)	2 (5.7%)
Borderline 2	66 (48.5%)	0 (0.0%)
Borderline 3	7 (5.1%)	0 (0.0%)

^c^ More than one diagnosis per patient possible

### Intercorrelation of STIPO dimensions

All STIPO domains correlated significantly with each other (all p-values < 0.001); correlations ranged from 0.39 to 0.78 (see Fig. S1 in the supplementary material).

### Categorical vs. dimensional PD diagnosis

All STIPO domains differed significantly between participants with a PD diagnosis in the IPDE and those without (*p* < 0.001). The group differences are demonstrated in Table 4.

**Table 4:** Difference in mean STIPO ratings by negative/ probably and definite PD (IPDE)

STIPO dimension	Mean (SD)		Test statistic		Effect size
	No IPDE diagnosis (*n* = 30)	IPDE diagnosis present (*n* = 141)	*t* (169)	*p*	Cohen’s *d*
Identity	1.43 (0.50)	3.57 (0.65)	17.062	< 0.001	3.43
Object relation	1.33 (0.55)	3.23 (0.70)	13.920	< 0.001	2.80
Primitive defenses	1.50 (0.63)	3.48 (0.64)	15.412	< 0.001	3.10
Coping rigidity	1.40 (0.56)	3.57 (0.70)	15.887	< 0.001	3.19
Self-directed aggression	1.60 (0.97)	3.91 (1.08)	10.797	< 0.001	2.17
Other-directed aggression	1.17 (0.46)	2.33 (0.93)	6.680	< 0.001	1.34
Moral values	1.23 (0.63)	2.39 (0.84)	7.167	< 0.001	1.44
Reality testing	1.13 (0.35)	2.53 (0.75)	9.951	< 0.001	2.00
Narcissism	1.20 (0.41)	2.90 (0.71)	12.504	< 0.001	2.63
Level of personality organization	0.70 (0.84)	3.53 (0.65)	20.585	< 0.001	4.13

We analyzed how much variance in the YSR total score could be explained by IPDE and STIPO outcome variables (see Supplement Table S1 for details). In the first regression model we observed that IPDE outcome explained 29.7% of variance in general psychopathology and adding STIPO outcome in the second step significantly increased the model fit by 17.6% (*p* < 0.001). The second regression model showed that STIPO outcome explained 43.4% of variance in the YSR total score; however, adding IPDE outcome in the second step did not significantly improve the model fit (Δ 3.9%; *p* = 0.723).

### Validation of the STIPO

We correlated the STIPO scores with instruments that are validated in adolescents, the IPO-A, the OPD-KJ2-SF the LoPF-Q, and the DAPP-BQ. Results are given in Table 5. Table 5 also demonstrates the correlations with psychopathology screened with the BDI (total score) and the YSR (total score, internalizing t-score, externalizing t-score). Correlations with the IPO-A were mostly significant in the domains “identity”, “aggression”, “reality testing” and “primitive defenses”. Opposed to our hypothesis, “identity diffusion” was not significantly correlated with STIPO “identity”, “aggression was not significantly correlated with STIPO “coping/rigidity” and “self-directed aggression”, and “moral values” were not significantly correlated with STIPO “identity”, “primitive defenses”, “coping/rigidity” and “self-directed aggression”. “Instability of goals” was not significantly associated with any STIPO domains. OPD-KJ2-SF and LoPF-Q had the highest correlations with all STIPO domains and overall STIPO ratings. The measures for psychopathology were significantly correlated with all STIPO domains. Correlations with the DAPP-BQ were smaller with significant associations of “dissocial behavior” and “emotion dysregulation” with all STIPO measures. “Inhibitedness” was significantly correlated to STIPO “object relation” only. “Compulsivity” was not significantly associated with any STIPO measures.

**Table 5:** Spearman correlations between STIPO domains and other questionnaire scales

		STIPO domains									
		Identity	Object relation	Primitive defense	Coping/rigidity	Self-directed aggression	Other-directed aggression	Moral values	Reality testing	Narcissism	STIPO level of personality organization
IPO	Identity diffusion	0.246	0.297*	0.364*	0.193	0.309*	0.385*	0.279*	0.444*	0.457*	0.341*
	Aggression	0.310*	0.377*	0.357*	0.231	0.188	0.517*	0.471*	0.403*	0.489*	0.418*
	Reality testing	0.299*	0.313*	0.350*	0.258*	0.313*	0.406*	0.354*	0.471*	0.407*	0.331*
	Primitive defense	0.378*	0.456*	0.469*	0.352*	0.419*	0.464*	0.416*	0.541*	0.532*	0.470*
	Moral values	0.257	0.275**	0.219	0.117	0.154	0.336*	0.336*	0.352*	0.348*	0.330*
	Instability of goals	− 0.181	− 0.041	− 0.114	− 0.125	− 0.134	− 0.051	− 0.109	− 0.110	− 0.216	− 0.106
OPD	Identity_t	0.617*	0.671*	0.647*	0.622*	0.513*	0.597*	0.507*	0.605*	0.566*	0.649*
	Control_t	0.635*	0.691*	0.696*	0.681*	0.552*	0.757*	0.509*	0.657*	0.649*	0.689*
	Interpersonality_t	0.635*	0.679*	0.663*	0.626*	0.566*	0.629*	0.487*	0.658*	0.546*	0.669*
	Attachment_t	0.603*	0.670*	0.633*	0.672*	0.572*	0.498*	0.501*	0.648*	0.550*	0.645*
	Total score_t	0.644*	0.707*	0.684*	0.670*	0.572*	0.657*	0.518*	0.666*	0.589*	0.687*
LoPF	Identity_t	0.691*	0.745*	0.712*	0.721*	0.634*	0.588*	0.579*	0.670*	0.636*	0.732*
	Self-direction_t	0.664*	0.694*	0.701*	0.699*	0.559*	0.598*	0.542*	0.620*	0.667*	0.709*
	Empathy_t	0.527*	0.567*	0.540*	0.497*	0.337	0.580*	0.525*	0.474*	0.518*	0.559*
	Intimacy_t	0.561*	0.668*	0.562*	0.580*	0.462*	0.410*	0.351*	0.463*	0.475*	0.594*
	Total score_t	0.671*	0.743*	0.694*	0.698*	0.566*	0.604*	0.551*	0.629*	0.630*	0.722*
Associations with measures of psychopathology											
	BDI total score	0.423*	0.528*	0.510*	0.442*	0.433*	0.341*	0.366*	0.481*	0.600*	0.514*
	YSR total t-score	0.384*	0.466*	0.468*	0.361*	0.381*	0.496*	0.404*	0.552*	0.524*	0.498*
	YSR internalizing t-score	0.368*	0.464*	0.441*	0.372*	0.346*	0.382*	0.291*	0.498*	0.507*	0.439*
	YSR externalizing t-score	0.308*	0.363*	0.373*	0.319*	0.292*	0.509*	0.489*	0.450*	0.470*	0.443*
DAPP	Dissocial behavior	0.322*	0.329*	0.364*	0.249*	0.255*	0.459*	0.444*	0.351*	0.487*	0.420*
	Inhibitedness	0.233	0.311*	0.225	0.191	0.220	0.183	0.128	0.218	0.290	0.215
	Emotional dysregulation	0.470*	0.533*	0.542*	0.455*	0.506*	0.534*	0.430*	0.586*	0.645*	0.563*
	Compulsivity	− 0.059	0.068	− 0.047	− 0.004	− 0.018	0.100	− 0.051	0.057	− 0.074	− 0.025

*p ≤ 0.001

### Sensitivity analyses: exploration of differences between STIPO versions

Forty-eight patients were rated with the original version of the STIPO, 88 patients and all controls were rated by the STIPO-R-A. We therefore explored whether the STIPO ratings gained from these two versions differed in any way. Indeed, we did not observe any difference between the average STIPO ratings between the versions (see Table S2 in the supplementary material). We further explored whether the correlational pattern between the STIPO domains (STIPO vs. STIPO-R-A) and IPO domains differed, and again did not observe a systematic difference between the versions (see Table S3 in the supplementary material).

## Discussion

The new diagnostic paradigm in PD focuses on impairment of personality functioning rather than the presence or absence of specific symptoms. Therefore, new assessment tools especially for adolescents are called for. This study was performed to observe the utility and validity of an assessment tool for the diagnosis of PD in adolescents, the STIPO. We included severely impaired adolescents, that had a categorical PD diagnosis as well as healthy adolescents. 97% of our patient sample presented with clinically relevant externalizing and internalizing psychopathology as well as depression. In that way, it is a unique study, and there is to the best of our knowledge only one other study including adolescents with and without PD that used a structured interview for dimensional assessment of PD [25].

Our results demonstrate that STIPO levels 3–5, i.e. mild, moderate and severe level of BPO, were significantly correlated with the categorical PD diagnosis in the IPDE. To address the question whether this dimensional approach is clinically useful and relevant, we correlated depressive, internalizing and externalizing symptoms with each STIPO domain and with the overall level of PO. Results confirmed that the severity level in the STIPO significantly correlated with broad measures of psychopathology. Furthermore, the STIPO significantly surpassed the informative value of the categorical IPDE outcome in explaining psychopathology variance as measured by the YSR, thereby emphasizing the diagnostic and clinical utility of this dimensional assessment of PD in adolescence. The dimensional assessment of the STIPO defines a severity grade as advised by national guidelines [19].

For validation purposes we compared each STIPO domain with several domains of validated questionnaires for adolescents and found significant correlations confirming our hypotheses. The IPO-A is a self-rating instrument that is based on the same theoretical concept, Kernberg’s object relation model [49]. Therefore, it was expected (hypothesis 1) that significant correlations were found in each domain and overall level of PO. Significant correlations of the STIPO domains with most IPO domains mainly confirm our hypothesis. Disturbed identity is a central criterion of impaired personality functioning, but the distinction of identity diffusion and identity crisis in adolescence is difficult. An interview process for its examination is recommended in contrast to self-rating questionnaires [25, 58, 59]. This limitation of the self-rating questionnaire might explain that the correlation of STIPO “identity” and IPO-A “identity diffusion” was not statistically significant. A significant positive correlation was found for IPO-A “primitive defenses”, in line with Kernberg´s theory, that primitive defenses based on splitting, e.g. projective identification, denial, idealization and devaluation, represent an important factor in the diagnostic process of adolescent PD. “Primitive defenses” might be a domain that is easier assessed with self-rating instruments, as proposed by others [47]. The overall STIPO level was significantly correlated to all IPO-A domains but “instability of goals”. This is in line with results from the validation study of the German version of the IPO-A, where it has also been shown that “instability of goals” was not associated with personality pathology [47]. “Instability of goals” might therefore not be a valid domain to distinguish adolescent crises from PD. The significant correlations of nearly all STIPO domains to the remaining IPO domains stressed out, that the STIPO and the IPO-A both assess the different manifestations of Kernberg´s concept on personality organization in line with studies in adults [43].

The second instrument, the OPD KJ2 SF, is a questionnaire that assesses the “structure” axis of the OPD [50]. It has been shown that the OPD “structure” dimension is aligned with the concept of personality functioning of the AMPD in the DSM-5 [60]. As hypothesized (hypothesis 2), the OPD total score was used for the validation of the German STIPO in adults [43] and correlated positively with the STIPO overall rating. In our study, all domains of the OPD KJ SF correlated significantly with each STIPO domain and with the level of PO adding evidence to the validity of the STIPO in adolescents.

As predicted (hypothesis 3), our results verify that the concept of PO assessed with the STIPO is aligned with the concept of personality functioning. The LoPF domains “identity”, “self-direction”, “empathy” and “intimacy” measuring the core elements of personality functioning correlated highly significantly with almost all STIPO domains. The LoPF is the only self-report measure that revealed good internal consistency and construct validity to assess personality functioning [61], therefore the correlation with STIPO domains strongly supports its validity and its usefulness in the light of the new classification systems.

All STIPO domains correlated significantly with each other underscoring that the domains are not independent and adding evidence to the accuracy of Kernberg’s conceptualization of PO [62]. The results are in line with the validation study of the German version of the STIPO for adults [43]. Authors highlighted the clinical value of the different domains that are manifestations of the same core pathology.

We used two different versions of the STIPO. Ratings as well as correlations between the IPO-A and the STIPO did not differ between these versions. We therefore suggest the use of the revised STIPO that was put into adolescent language by the study team (STIPO-R-A) for assessment in adolescents since it was easier to understand by the patients and less time consuming without reducing its meaningfulness.

After assessing personality functioning (Criterion A of the AMPD of the DSM-5 and ICD-11), Criterion B of the AMPD and the ICD-11 specify pathological personality traits. Maladaptive traits define the type of PD as they indicate what kind of problems the patient might have [37]. Five domains are listed that are aligned with the Five Factor Model of personality and assessment inventories have been developed [33]. The DAPP-BQ [34] that is validated in adolescents [35] was reported to have a high overlap with the Five Factor Questionnaires established for the ICD-11 [36]. As predicted (hypothesis 4), “dissocial behavior”, representing a lack of regard for others and “emotional dysregulation”, representing unstable tendencies, dissatisfaction with the self and life experiences, and interpersonal problems, were traits that positively correlated with all STIPO domains and the overall level of PO. Significant correlations were smaller than correlations with measures of personality functioning. This was expected as the two constructs are different. “Dissocial behavior” had highest correlations with “other directed aggression”, “moral values”, “narcissism” and overall STIPO level. This suggests that impaired moral values and narcissistic dysregulation is prominent in patients with low PO supporting the conceptualization of pathological narcissism and dissocial behavior by O. Kernberg [63]. Therefore, “moral values” and “narcissism” are domains that might be helpful for severity staging. “Emotional dysregulation” was highly correlated to all STIPO domains and might refer to an overarching problem in PD. Emotion regulation has also been proposed as transdiagnostic construct and might therefore, especially in adolescence, be a rather unspecific marker for psychopathology as well as for impaired personality functioning [64]. “Inhibitedness”, representing little enjoyment from intimate relationships, was significantly correlated to STIPO “object relation”. The results are line with the before mentioned study that outlined the predictive capacity of these personality traits [36]. Authors reported that “inhibitedness” predicted Cluster A, “dissocial behavior” and “emotional dysregulation” predicted Cluster B and “emotional dysregulation” only Cluster C PDs. In line with this study, the present study found that personality trait “compulsivity” was the only trait not significantly correlated with the domains of the STIPO. This trait might not play a key role in adolescent PD and further investigation is needed to shed light on personality functioning and “compulsivity” trait pathology.

All STIPO domains and level of PO were significantly positively associated with adolescent psychopathology assessed with the YSR and the BDI (hypothesis 5). Our results are in line with the work by Thomson et al. that revealed coinciding impairments of self-functioning and depressive symptoms as well as higher severity of psychopathology [25].

### Clinical implications

The STIPO dimensions “identity” and “object relations” reflect Criterion A of the AMPD in the DSM-5 for diagnosing PD in adolescents. “Coping” and “primitive defense mechanisms” are closely connected to impaired personality functioning from a psychodynamic viewpoint and are of importance for treatment considerations [65]. “Self- and other-directed aggression” refers to nonsuicidal self-injuries and suicidality that are prioritized treatment targets in adolescent PD [66]. “Moral values” is a meaningful domain that is important to consider, as PD patients with rigid moral values or dissocial behavior need special treatment and manipulative behavior might undermine therapeutic interventions [67]. “Reality testing” for the assessment of derealization and depersonalization as well as to detect psychotic symptoms, e.g. hallucinations, is of importance as it is considered a marker of clinical severity and impairments in this dimension hampers treatment adherence [68, 69]. From a clinical point of view, consideration of these domains helps in treatment planning [70]. Especially for longitudinal investigations of adolescent PD through emerging adulthood it seems highly beneficial that the STIPO-R-A and the adult revised version of the STIPO (STIPO-R) are comparable. Future outcome research should also focus on improvement of personality functioning and not only on symptom reduction.

While the study has some strengths, the results also have to be interpreted in the light of some limitations. A strength is the patient sample size within a so far under-researched population, especially considering that the patients included had a high symptom load and all had a PD diagnosis based on a valid clinical interview. Though small in sample size, we included healthy adolescents that enabled us to encompass the whole spectrum from normal to neurotic to borderline PO. The interviews were conducted by highly trained professionals with experience to consider the developmental phase of adolescence. To obtain a larger sample size, we decided to include 48 patients that were rated with the original STIPO version, done in a time when the revised version was not yet published. Although the revised version is mainly a reduction of redundant items and should therefore be comparable with the original STIPO, we performed a sensitivity analysis. It showed no differences of STIPO and STIPO-R outcome, but it has to be kept in mind that the analysis was done comparing the first 48 patients with the following 88 patients. The majority of adolescents in our study sample was female which limits the generalizability of our results. In clinical settings, female adolescents are overrepresented as seen in other studies, e.g [25, 68]. and possibly due to that fact, female patients are focused on in research of PD [71]. Whether the prevalence regarding sex is different is still matter of debate [72]. A recent review paper suggests no prevalence difference, but different symptomatology with females presenting with internalizing and males with externalizing symptoms [73]. Studies in male adolescents are necessary to fill this research gap. Additionally, longitudinal assessment of PO would be necessary to encompass the impact of BPO on psychosocial functioning and development through emerging adulthood as well as addressing the question concerning stability of the diagnosis. Furthermore, studies on treatment and treatment outcome should not only monitor and report on symptom reduction but also improvement of personality functioning.

## Conclusion

Our study indicates that the STIPO-R-A is a reliable and valid instrument for the assessment of PD in adolescents that can validly differentiate between adolescent identity crises and PD. It comprises the core elements of personality functioning, as requested in Criterion A in the AMPD of the DSM-5 and the ICD-11 and is useful for early detection and staging of severity in adolescents. Its clinical value goes beyond the dimensions of identity and object relations. Underlying defense mechanisms, the severity of self- and other-directed aggression, moral values and reality testing, as assessed by the STIPO, are considered to be useful for treatment planning as well as for prognostic considerations.

## Electronic supplementary material

Below is the link to the electronic supplementary material.

Supplementary Material 1

## Data Availability

No datasets were generated or analysed during the current study.

## Abbreviations

AMPD: Alternative model for personality disorders
BDI: Beck´s Depression inventory
BPO: Borderline personality organization
DAPP-BQ: Dimensional assessment of personality pathology-basic questionnaire
HC: Healthy control
ICC: Interclass correlation coefficients
IPDE: International personality disorder examination
IPO-91-A: Inventory of personality organization-adolescents
LoPF-Q: Levels of personality functioning questionnaire
OPD-KJ2-SF: Operationalised psychodynamic diagnosis in children and adolescents - structure questionnaire
PD: Personality disorders
PO: Personality organization
STIPO: Structured interview for personality organization
STIPO-R: Structured interview of personality organization, revised version
STIPO-R-A: Structured interview of personality organization, revised version, adapted to the adolescent language
YSR: Youth self-report
