# Supplementary material for: Assessment of personality disorders in adolescents – a clinical validity and utility study of the structured interview of personality organization (STIPO)
Source: Child Adolesc Psychiatry Ment Health. 2025 May 2;19:49. doi: 10.1186/s13034-025-00901-9 (PMC12049018; doi:10.1186/s13034-025-00901-9)
Supplement: Supplementary file 1 — Supplementary Material 1 [file 13034_2025_901_MOESM1_ESM.docx]

**Supplementary material**

**Figure S1.** Spearman correlations between STIPO dimensions (Note: All correlation coefficients are statistically significant p < .001)


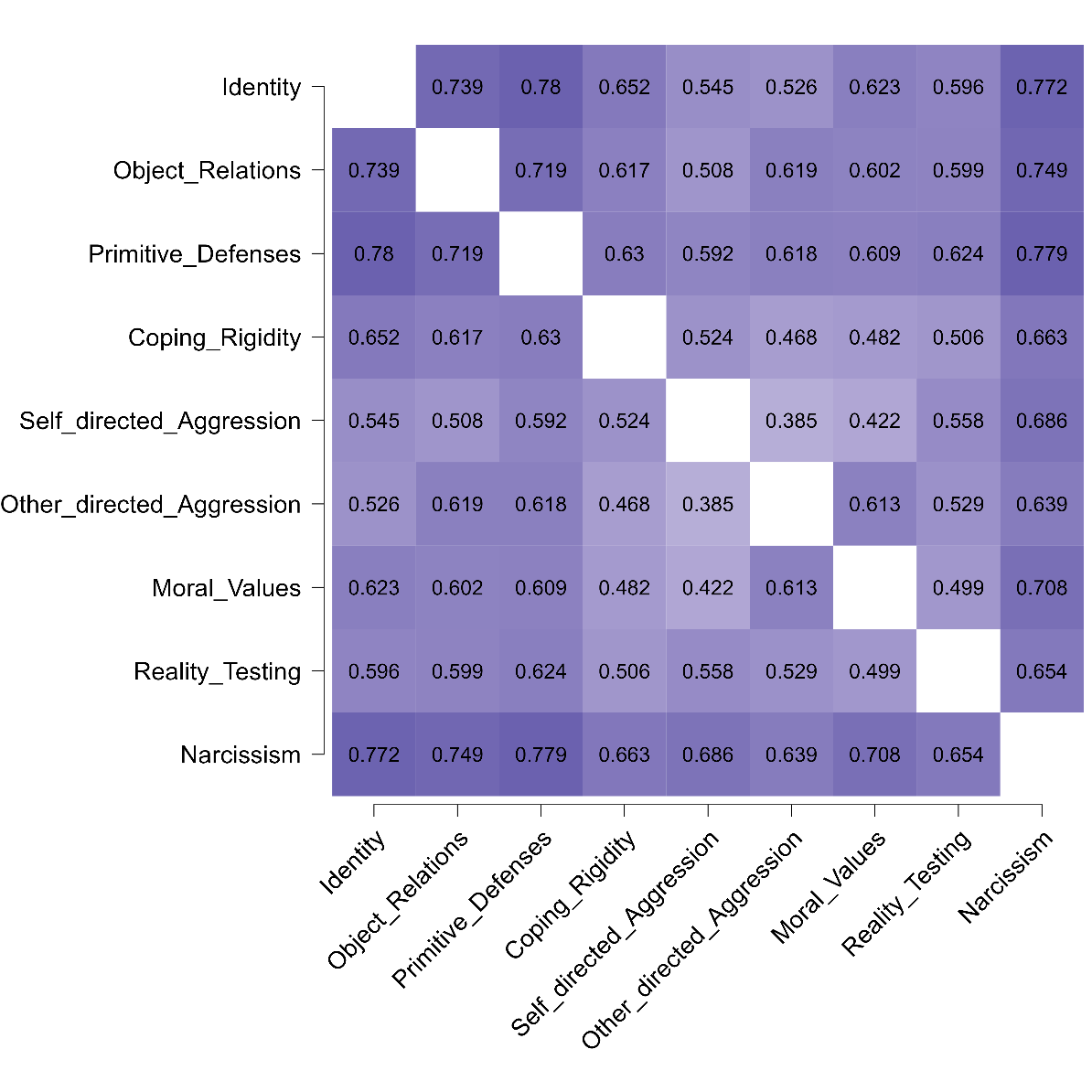


**Table S1.** Results of hierarchical linear regression models predicting general psychopathology (YSR total T-score) by IPDE and STIPO outcomes

| Model 1 | *R* | *R*² | Δ*R*² | *p* |
| --- | --- | --- | --- | --- |
| Step 1 (IPDE outcomes)^a^ | .545 | .297 |  |  |
| Step 2 (STIPO outcomes)^b^ | .688 | .473 | .176 | < .001 |
| Model 2 | *R* | *R*² | Δ*R*² | *p* |
| Step 1 (STIPO outcomes)^b^ | .659 | .434 |  |  |
| Step 2 (IPDE outcomes)^a^ | .688 | .473 | .039 | .723 |

^a^ includes all IPDE diagnostic outcomes (paranoid, schizoid, schizotypal, antisocial, borderline, histrionic, narcissistic, avoidant, dependent, obsessive-compulsive each coded as 0 = none, 1 = probably, 2 = definite).

^b^ includes the following STIPO outcomes: identity, object relations, primitive defenses, coping rigidity, self-directed aggression, other-directed aggression, moral values, reality testing, narcissism (each coded from 1-5)

**Table S2.** Differences in mean STIPO ratings between STIPO versions among patients

|  | STIPO  (N = 48) | STIPO-R-A  (N = 88) | *t*(134) | *p* |
| --- | --- | --- | --- | --- |
|  | Mean (SD) | Mean (SD) |  |  |
| Identity | 3.69 (0.62) | 3.58 (0.60) | 0.987 | .325 |
| Object relation | 3.17 (0.72) | 3.32 (0.67) | -1.224 | .223 |
| Primitive defense | 3.46 (0.62) | 3.53 (0.62) | -0.679 | .498 |
| Coping / rigidity | 3.65 (0.64) | 3.58 /0.69) | 0.550 | .583 |
| Self-directed aggression | 3.71 (0.99) | 4.07 (1.08) | -1.911 | .058 |
| Other-directed aggression | 2.35 (0.91) | 2.38 (0.94) | -0.125 | .901 |
| Moral values | 2.38 (0.82) | 2.44 (0.84) | -0.456 | .649 |
| Reality testing | 2.46 (0.82) | 2.61 (0.70) | -1.159 | .249 |
| STIPO level of personality organization | 3.48 (0.58) | 3.63 (0.63) | -1.323 | .188 |

**Table S3.** Spearman correlations between STIPO domains and IPO outcomes by STIPO versions (patients only)

|  | STIPO domains | | | | |  | | | | |
| --- | --- | --- | --- | --- | --- | --- | --- | --- | --- | --- |
|  | Identity | Object relation | Primitive defense | Coping / rigidity | Self-directed aggression | Other-directed aggression | Moral values | Reality testing | STIPO level of personality organization |  |
| ipo_Identity diffusion | -.02/.17 | .22/.06 | .22/.27* | -.02/.01 | .09/.26* | .34*/.28* | .23/.21 | .42**/.36** | .18/.25* |  |
| ipo_Aggression | .09/.35** | .21/.29* | .17/.26* | -.19/.17 | -.03/.12 | .52**/.37** | .51**/.43** | .55**/.29* | .22/.46** |  |
| ipo_Reality testing | -.12/.20 | .18/.03 | .01/.26* | -.08/.07 | .09/.18 | .43**/.21 | .23/.26* | .55**/.27* | .07/.15 |  |
| ipo_Primitive defenses | .09/.01 | .22/.07 | .20/.15 | .03/-.03 | .17/.13 | .27/.22 | .22/.18 | .47**/.25 | .19/.11 |  |

*Note:* First correlation coefficient represents the correlation in the old STIPO version. the second correlation coefficients represent the correlation in the new STIPO version;

* *p* < .05, ** *p* < .01
